# Endometrium-Limited Metastasis of Extragenital Malignancies: A Challenge in the Diagnosis of Endometrial Curettage Specimens

**DOI:** 10.3390/diagnostics10030150

**Published:** 2020-03-10

**Authors:** Sangjoon Choi, Jin Woo Joo, Sung-Im Do, Hyun-Soo Kim

**Affiliations:** 1 Department of Pathology and Translational Genomics, Samsung Medical Center, Sungkyunkwan University School of Medicine, Seoul 06351, Korea; choisj88@gmail.com; 2 Department of Pathology, Severance Hospital, Yonsei University College of Medicine, Seoul 03722, Korea; darkjoogga@hanmail.net; 3 Department of Pathology, Kangbuk Samsung Hospital, Sungkyunkwan University School of Medicine, Seoul 03181, Korea; sungim.do@samsung.com

**Keywords:** endometrium, metastasis, extragenital malignancy, immunohistochemistry

## Abstract

Malignancies of extragenital origin very rarely metastasize to the uterine body. Endometrium-limited metastases may pose diagnostic challenges in endometrial curettage specimens as they may be misdiagnosed as primary endometrial tumors. We investigated the clinicopathological characteristics of seven cases with endometrial-limited metastases from carcinomas of the nasopharynx (*n* = 1), breast (*n* = 2), colon (*n* = 2), stomach (*n* = 1), and appendix (*n* = 1). The patients’ ages ranged from 36 to 71 (mean: 55.4) years. None of the patients had a remarkable gynecological history, and the presenting sign in all cases was abnormal uterine bleeding. Although myometrial involvement was absent, multiple metastases were already present in extrauterine locations such as the lung, liver, bone, abdominopelvic peritoneum, and omentum. All patients underwent ultrasonographic examination prior to endometrial curettage. The histologies of the endometrial metastases identified from the curettage specimens were identical to those of the corresponding primary tumors. Ancillary tests including immunostaining and Epstein–Barr virus-encoded RNA in situ hybridization confirmed the extragenital origin. Endometrium-limited metastases from extragenital malignancies are extremely rare. They present with abnormal vaginal bleeding and mimic endometrial carcinomas of endometrioid or poorly differentiated types. Since their clinical presentations and histological features are similar to those of primary endometrial tumors, pathologists should consider the possibility of metastases while evaluating endometrial curettage specimens obtained from patients with a history of extragenital malignancies.

## 1. Introduction

Metastases to female reproductive organs often pose diagnostic challenges for both pathologists and clinicians, and tend to be overlooked in the differential diagnoses of other gynecological malignancies. In the rare event of metastases to the female genital tract, the ovaries are most often affected [1]. Malignancies that secondarily involve the uterus usually extend directly from adjacent pelvic organs including the rectum, urinary bladder, and peritoneum; alternatively, they may develop consequent to widespread peritoneal dissemination. Embolic metastases to the uterine body are rare, particularly from extragenital malignancies. Numerous studies have investigated various aspects of metastatic tumors to the female reproductive organs [2,3,4,5,6,7,8,9,10,11]. However, to the best of our knowledge, only few case series of endometrial metastases from extragenital malignancies are available in literature [3,4,6,12,13]. Information of diagnostic utility is conspicuously lacking among previous studies in literature and standard textbooks [2,3,4,5,6,7,14].

The myometrium is the part of the uterus that is most commonly involved by metastatic tumors [15,16,17]. Endometrial involvement by metastasis almost always occurs in conjunction with myometrial metastasis. However, we recently encountered a very rare case of endometrium-limited metastasis from non-keratinizing undifferentiated carcinoma of the nasopharynx; this initiated a comprehensive review of our archival cases. In this study, we investigated the clinicopathological characteristics and results of ancillary tests in seven cases of endometrial metastases from extragenital organs including the nasopharynx, breast, stomach, colon, and appendix. Endometrial metastases are believed to be extremely rare, and may easily be misdiagnosed as primary endometrioid or poorly differentiated carcinomas of the endometrium, rather than metastatic disease. This comprehensive analysis of these unusual cases will serve to improve the current understanding of endometrial metastases from extragenital malignancies.

## 2. Materials and Methods

### 2.1. Case Selection

This study (4-2017-1148) was reviewed and approved by the Institutional Review Board of the Severance Hospital (Seoul, Republic of Korea), approval date 24 January 2018, and the cases were selected from the computerized archival files. Written informed consent for publication of the article could not be obtained because five of the seven patients were dead and the remaining two patients were lost to follow-up. Cases where the primary tumors originated in the female genital tract, including the uterine cervix and body, ovaries, and salpinges, and those where the uterus was invaded by contiguous spread from adjacent pelvic organs, were excluded. Leukemic infiltrates and malignant lymphomas were also excluded. Cases with metastases to both the endometrium and myometrium were excluded. During the five-year study period from March 2013 to February 2018, seven patients were diagnosed with endometrium-limited metastatic carcinomas of extragenital origin. Clinical and pathological data including age at diagnosis, previous gynecological history, presenting symptoms, site and histological subtype of primary tumors, location of extrauterine metastases, treatment, and current status, were obtained from the electrical medical information systems and pathology reports.

### 2.2. Pathological Examination

Pathologists initially examined the resected tissues prior to fixation in 10% neutral-buffered formalin. The tissues were thoroughly examined macroscopically after fixation for 12–24 h, and were then sectioned. After processing using an automatic tissue processor (Peloris II, Leica Microsystems, Wetzlar, Germany), the sections were embedded in paraffin blocks; 4 µm-thick slices were prepared from each formalin-fixed paraffin-embedded tissue (FFPE) block, using a rotary microtome (RM2245, Leica Microsystems). The sections were then stained with hematoxylin and eosin using an automatic staining instrument (Ventana Symphony System, Ventana Medical Systems, Tucson, AZ, USA). After staining, the slides were covered with a glass coverslip and were examined by board-certified pathologists specialized in gynecological oncology. Definitive pathological diagnoses were made after examination of all available hematoxylin and eosin-stained slides by light microscopy (BX43 System Microscope, Olympus, Tokyo, Japan); the most representative slide was selected for performing immunohistochemical staining and/or Epstein–Barr virus-encoded RNA in situ hybridization (EBER-ISH).

### 2.3. Immunohistochemistry

The 4 μm-thick, FFPE slices were de-paraffinized and rehydrated using a xylene and alcohol solution. Immunostaining was performed using automated instruments (Ventana Benchmark XT (Ventana Medical Systems) and or Dako Omnis (Dako, Carpinteria, CA, USA)) [1,18,19,20,21,22,23,24,25,26]. After antigen retrieval, the slices were incubated with primary antibodies including pan-cytokeratin (pan-CK; 1:600, clone AE1/AE3, Dako), CK7 (Dako, 1:100, clone OV-TL 12/30, Dako), CK20 (1:100, clone Ks20.8, Dako), caudal type homeobox 2 (CDX2; 1:400, clone EPR2764Y, Cell Marque, Rocklin, CA, USA), paired box 8 (PAX8; 1:50, polyclonal, Cell Marque), estrogen receptor (ER; 1:150, clone 6F11, Novocastra, Leica Biosystems, Newcastle Upon Tyne, UK), progesterone receptor (PR; 1:100, clone 16, Novocastra), p53 (1:300, clone DO-7, Novocastra), p16 (prediluted, clone E6H4, Ventana Medical Systems), p63 (1:50, clone 4A4, Dako), and GATA-binding protein 3 (GATA3; 1:150, clone L50-823, Cell Marque). After chromogenic visualization, the slices were counterstained with hematoxylin. Appropriate positive and negative controls were concurrently stained to validate the staining method [27,28,29]. Negative controls were prepared by substituting non-immune serum for primary antibodies, resulting in no detectable staining.

### 2.4. EBER-ISH

The tissue sections obtained from the endometrial curettage specimens were used for EBER-ISH. The sections were de-paraffinized with xylene, pretreated with proteinase K for 20 min, and incubated with fluorescein isothiocyanate-conjugated Epstein-Barr virus (EBV)-encoded RNA oligonucleotide probes (Novocastra) at 55 °C for 2 h. The sections were then rinsed in water and incubated with horseradish peroxidase-conjugated anti-fluorescein isothiocyanate antibody for 15 min before adding the chromogen to produce an alcohol-insoluble dark nuclear stain in the EBV-positive cells. We used EBV-absent lymphoid tissues processed using the hybridization mixture without EBV-encoded RNA oligonucleotides, as negative controls [30].

## 3. Results

### 3.1. Clinical Characteristics

**Figure 1:**
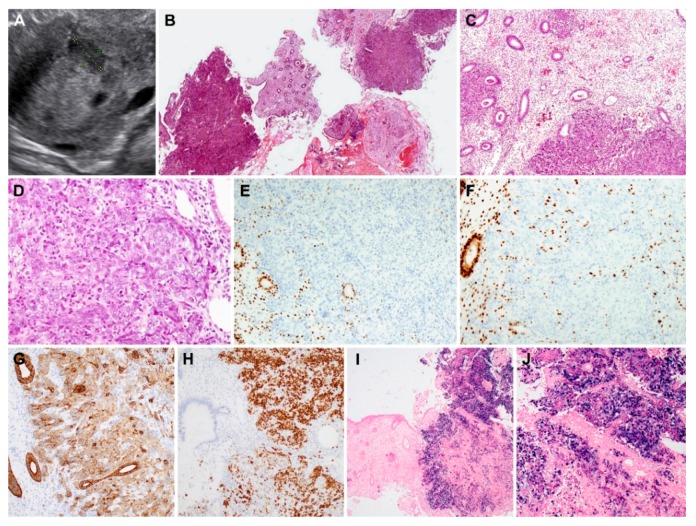
Histopathological findings, immunostaining results, and Epstein–Barr virus-encoded RNA in situ hybridization (EBER-ISH) results of metastatic non-keratinizing undifferentiated carcinoma of the nasopharynx (case 1). (**A**) Abdominopelvic ultrasonography reveals a hypoechoic endometrial mass. (**B**) On scanning view, several endometrial tissue fragments are totally or partially replaced by tumor tissues. (**C**) Low-power magnification shows variable-sized tumor cell clusters infiltrating the endometrial stroma. The glands are not involved; however, they are surrounded by tumor cells. (**D**) High-power magnification exhibits an admixture of tumor cells showing syncytial growth and polymorphic inflammatory cells. The tumor cells possess large pleomorphic nuclei with conspicuous nucleoli. (**E**,**F**) Tumor cells are negative for (**E**) estrogen receptor (ER) or (**F**) progesterone receptor (PR); in contrast, these proteins are uniformly and strongly expressed in the nuclei of endometrial glandular epithelium and stromal cells. (**G**) Intensity of CK7 immunoreactivity is strong and moderate in the endometrial glandular epithelium and tumor cells, respectively. (**H**) Tumor cells display strong nuclear p63 expression. (**I**) EBER-ISH reveals positive signaling in tumor tissues (right). (**J**) Higher magnification of image (**I**) Tumor cells exhibit strongly positive nuclear staining. Original magnification: **B**: ×12.5; **C**: ×40; **D**: ×200; **E**–**H**: ×100, **I**: ×12.5; **J**: ×40.

Table 1 summarizes the clinical characteristics. The patients’ ages ranged from 36 to 71 (mean: 55.4) years. None of the patients had a history of gynecological disease, and all had abnormal uterine bleeding as the presenting sign. Four patients had been diagnosed with carcinomas of gastrointestinal origin, including those of the colon, appendix, and stomach. Two and one patients had breast and nasopharyngeal carcinomas, respectively. Imaging findings including computed tomography (CT) and magnetic resonance imaging (MRI) scans revealed no metastatic lesions involving the myometrium, parametrium, and uterine serosa, i.e., the endometrium was the only site of metastasis in the uterus. However, all patients had already developed multiple metastases outside the uterus including the ovaries, lymph nodes, lungs, liver, bone, adrenal glands, retroperitoneum, omentum, abdominopelvic peritoneum, and abdominal wall. Except for one patient who refused further treatment and was lost to follow-up, all others had received chemotherapy. Four of the six patients whose follow-up data were available experienced progressive disease, and the remaining two had died. The seven cases are presented below:

Case 1. A 44-year-old woman with no previous gynecological history presented with abnormal uterine bleeding since the past year. Biopsy of a nasopharyngeal mass two years previously had revealed a diagnosis of a non-keratinizing undifferentiated carcinoma. MRI of the neck showed a 2 cm-sized mass in the left nasopharynx, and multiple enlarged lymph nodes in the left retropharyngeal and supraclavicular areas (stage IVB). She had undergone adjuvant chemotherapy after being diagnosed with nasopharyngeal carcinoma. Positron emission tomography-computed tomography (PET-CT) and ultrasonography were performed to identify the source of bleeding. Whole body PET-CT revealed multiple foci of intense fluorodeoxyglucose uptake in the liver, adrenal gland, bone, and endometrium. Abdominopelvic ultrasonography revealed a homogenous hypoechoic lesion in the endometrium measuring 1.1 cm (Figure 1A); bilateral ovarian masses were also noted. An endometrial curettage was performed based on the initial impression of a primary endometrial lesion. She is currently receiving chemotherapy for progression of hepatic metastases.

Case 2. A 60-year-old woman with a history of breast carcinoma presented with abnormal uterine bleeding. She underwent total mastectomy with axillary lymph node dissection for invasive lobular carcinoma of the breast 3 years previously. Immunohistochemically, the tumor was diffusely and strongly positive for ER and PR, but negative for human epidermal growth factor receptor 2 factor (HER2). Whole body PET-CT revealed suspected metastatic lymph nodes in the neck and axilla, bilateral ovaries, axial and bilateral pelvic bones, liver, and retroperitoneum. A diagnostic endometrial curettage was performed to determine the cause of uterine bleeding.

Case 3. A 47-year-old woman presented with abnormal uterine bleeding and a small nodule in the scalp. She was diagnosed with invasive ductal carcinoma of the left breast 4 years previously. Immunostaining revealed that the tumor was negative for ER, PR, and HER2, compatible with triple-negative breast carcinoma (TNBC). She underwent left partial mastectomy with axillary lymph node dissection, and received adjuvant chemotherapy and radiation therapy for lymph node metastases. She also underwent wedge resection of the right lung for multiple lung metastases two years previously. An endometrial curettage was performed to determine the cause of uterine bleeding. She is currently receiving chemotherapy for progression of lung metastases.

Case 4. A 71-year-old woman was diagnosed with a primary adenocarcinoma of the transverse colon with synchronous multiple hepatic metastases two years previously; she underwent neoadjuvant chemoradiation therapy and extended right hemicolectomy, and had also received radiation therapy for multiple bone metastases. A subsequent CT of the chest revealed multiple lung nodules and bilateral pleural effusions, and the carcinoembryonic antigen level in the pleural fluid was markedly elevated (1232 ng/mL). She presented with abnormal uterine bleeding one year previously. Although abdominopelvic ultrasonography revealed multiple uterine intramural leiomyomata, a diagnostic endometrial curettage was performed owing to the possible presence of invisible lesions in the endometrium. She developed fever and leukocytosis during chemotherapy for multiple abdominopelvic peritoneal metastasis and died of infection.

Case 5. A 67-year-old woman was diagnosed with a primary adenocarcinoma of the rectosigmoid colon and underwent low anterior resection and subsequent adjuvant chemotherapy. After two years postoperatively, metastatic tumors were detected in the bilateral ovaries, abdominal wall, liver, and bilateral lungs. In addition, an endometrial curettage specimen revealed metastatic colorectal adenocarcinoma. Despite a second course of chemotherapy, she died 8 months later with disseminated disease.

Case 6. A 63-year-old woman was presented with abnormal uterine bleeding. Abdominopelvic ultrasonography revealed no endometrial lesion. However, the diagnosis of metastatic signet ring cell carcinoma was made in the endometrial curettage specimen. MRI revealed mild diffuse peritoneal infiltration, suspicious for early peritoneal carcinomatosis. Diagnostic laparoscopy with appendectomy was performed. The appendix had signet ring cell carcinoma. She refused further work-up and treatment, and was lost to follow-up.

Case 7. A 36-year-old woman was diagnosed with signet ring cell carcinoma of the gastric cardia, two years previously. Diffuse metastatic dissemination was detected in the abdominopelvic peritoneum and omentum, one year after chemotherapy. During palliative chemotherapy, she presented to the emergency department with abnormal uterine bleeding. Ultrasonography revealed no definite mass lesions in the endometrium and myometrium. An endometrial curettage was performed to determine the cause of uterine bleeding.

### 3.2. Pathological Characteristics

The histological subtypes of the primary tumors included nasopharyngeal non-keratinizing undifferentiated carcinoma, invasive lobular and ductal carcinomas of the breast, colonic adenocarcinomas, appendiceal signet ring cell carcinomas, and gastric signet ring cell carcinomas. The metastatic tumors of the endometrium demonstrated identical histological features as those of the corresponding primary tumors. The histological findings obtained from the endometrial curettage specimens are described below:

Metastatic nasopharyngeal carcinoma (case 1). The curetted specimen comprised fragmented proliferative endometrium with several large irregular-shaped clusters and sheets of tumor cells (Figure 1B). The tumor cells were arranged in syncytial pattern without distinct cell borders (Figure 1C) and were admixed with inflammatory cells within the clusters and sheets that predominantly comprised lymphocytes, plasma cells, and some eosinophils. The tumor cells possessed moderate eosinophilic cytoplasm, and large round nuclei with single or multiple conspicuous eosinophilic nucleoli (Figure 1D). The mitotic activity was markedly increased (up to 16 per 10 high-power fields), and atypical mitotic figures were often identified. Based on the previous history, the histological findings were consistent with those of metastatic nasopharyngeal non-keratinizing undifferentiated carcinoma; however, the possibility of primary endometrial carcinomas of the poorly differentiated endometrioid or serous types could not be excluded. On immunostaining, the tumor cells were found to be negative for PAX8; however, they were positive for pan-CK (Figure 1G) and p63 (Figure 1H). The lack of ER (Figure 1E) and PR (Figure 1F) expression did not support a diagnosis of endometrioid carcinoma, and the patchy p16 and wild-type p53 expression patterns excluded serous carcinomas. EBER-ISH revealed diffuse and strong positivity in the tumor cells (Figure 1I,J).

**Figure 2:**
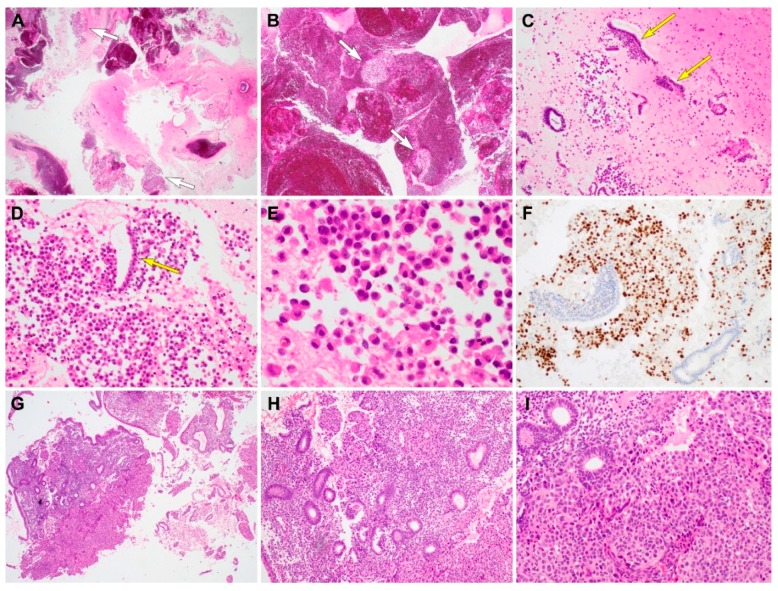
Histopathological findings and immunostaining results of metastatic invasive lobular (case 2) and ductal (case 3) carcinoma. (**A**,**B**) On scanning view, the curetted specimen consists mainly of blood and fibrin, and a small amount of scattered tissue fragments (short white arrows). (**C**) Low-power magnification shows individual tumor cells and endometrial strips (long yellow arrows) dispersed in the background of a fibrinous exudate. (**D**) Medium-power magnification demonstrates a monotonous population of discohesive tumor cells. A long yellow arrow indicates the endometrial strip located between the tumor cells. (**E**) On high-power magnification, the tumor cells appear round to polygonal and possess eccentrically placed hyperchromatic nuclei and eosinophilic cytoplasm, morphologically compatible with invasive lobular carcinoma of the breast. (**F**) Tumor nuclei are strongly positive for GATA3, whereas the endometrial strips do not express GATA3. (**G**) Metastatic carcinoma cells infiltrating the endometrial stroma. (**H**) Presence of benign endometrial glands, which are entrapped within tumor cells clusters, favors the diagnosis of a metastatic lesion. (**I**) Cohesive tumor cells forming solid sheets and a few small glands, demonstrating morphological compatibility with invasive ductal carcinoma of the breast. They possess large pleomorphic nuclei and abundant eosinophilic cytoplasm. Original magnification: **A** and **B**: ×12.5; **C**: ×40; **D**: ×40; **E**: ×200; **F**: ×40; **G**: ×12.5; **H**: ×40, **I**: ×100.

Metastatic invasive lobular (case 2) and ductal (case 3) carcinoma originating from the breast. The curetted specimens were paucicellular and consisted mainly of blood and fibrin, and a small amount of scattered tissue fragments and individual cells (Figure 2A,B). The fragmented endometrial glandular epithelia were separated from the stroma, resembling a glandular and stromal breakdown pattern. The cells of invasive lobular carcinoma were small to medium in size and were relatively uniform and round (Figure 2C,D). They had round nuclei with inconspicuous nucleoli, and a broad eosinophilic or vacuolar cytoplasm (Figure 2E). Although most of the invasive lobular carcinoma cells were dispersed individually among fragmented glandular epithelia, some were attached to small endometrial strips. Nuclear pleomorphism was minimal, and mitotic activity was low. Immunohistochemically, the tumor cells expressed ER, PR, and GATA3 (Figure 2F); however, PAX8 was not expressed. Diffuse and strong nuclear GATA3 immunoreactivity in the tumor cells that replaced the endometrial stroma, indicated a metastatic carcinoma originating from the breast. The cells of invasive ductal carcinoma were large and epithelioid, with granular eosinophilic or amphophilic cytoplasm. Their nuclei were clearly atypical, with prominent nucleoli and frequent mitotic figures. Several large anastomosing nests and sheets of tumor cells had replaced the endometrial stroma (Figure 2G). Most of the endometrial glands were loosely distributed within the stroma; however, some benign, inactive endometrial glands were partially or entirely entrapped by the tumor cells (Figure 2H). They demonstrated predominantly solid architecture, with a few foci of cribriform architecture and tubule formation (Figure 2I). Immunostaining revealed that the tumor cells were negative for ER, PR, and HER2. The histological features and immunophenotype were compatible with those of TNBC.

**Figure 3:**
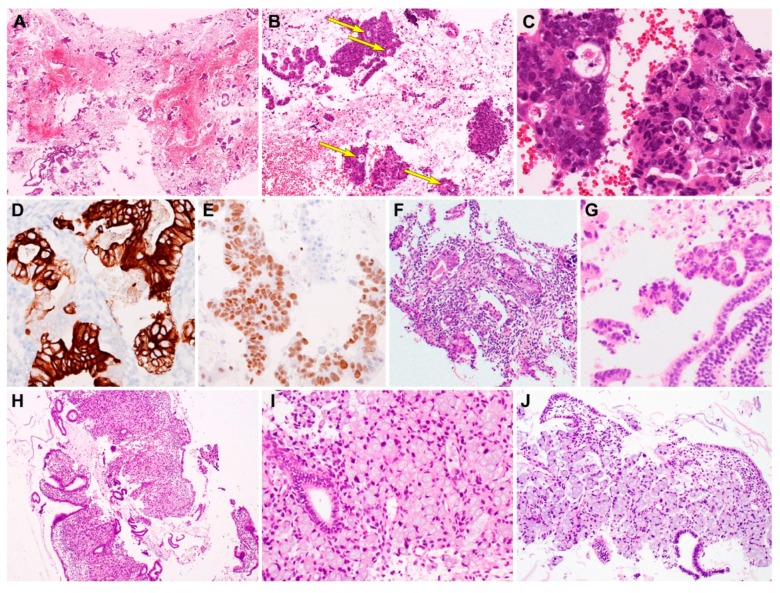
Histopathological findings and immunostaining results of metastatic gastrointestinal carcinoma. Metastatic colorectal adenocarcinoma (case 4). (**A**) Curetted specimen consists mainly of blood, fibrin, and necrotic debris admixed with tumor tissue fragments of variable size. (**B**) Irregular-shaped clusters of tumor cells exhibit solid and cribriform architecture with small glandular lumina (long yellow arrows). (**C**) Intraluminal eosinophilic material, necrotic debris, and severe nuclear pleomorphism of the glandular epithelium are morphologically consistent with colorectal adenocarcinoma. (**D**) Tumor cells are strongly positive for CK20. (**E**) Tumor cell nuclei also react uniformly with CDX2. Metastatic colorectal adenocarcinoma (case 5). (**F**) In a few foci, infiltrating tumor tissues are associated with small amount of stromal desmoplasia and artifactual clefts. (**G**) Fragmented epithelium possess larger, more hyperchromatic tumor cell nuclei, compared with those of endometrial strips (right lower corner). Metastatic gastric (case 6) and appendiceal (case 7) signet ring cell carcinoma. (**H**) Metastatic signet ring cell carcinoma from the stomach involving the endometrial stroma. (**I**,**J**) Medium-power magnification demonstrating aggregates of signet ring cells with typical morphological features of large collections of intracytoplasmic mucin compressing the nuclei towards the periphery of the cell. The entrapped endometrial glands possess small and bland nuclei. The uninvolved endometrial stroma display bland-appearing spindle cells (right lower corner). Original magnification: **A**, ×12.5; **B**, ×40; **C**: ×200; **D**: ×200; **E**, ×200; **F**, ×100; **G**, ×200; **H**, ×40; **I**, ×200; **J**, ×100.

Metastatic colorectal adenocarcinoma (case 4 and 5). Since both the patients with colorectal adenocarcinoma were postmenopausal, the curetted specimens had blood, mucin and small amounts of viable tissue (Figure 3A). Several scattered endometrial strips of variable sizes were found with interspersed irregular epithelial fragments. They displayed solid and cribriform architectural patterns, with intraluminal necrotic debris and inflammatory cells (dirty necrosis; Figure 3B). In some foci, the tumor cells infiltrated the endometrial stroma, and their nuclei were two- to three-fold larger than those of the adjacent endometrial glandular epithelial cells. The tumor cells possessed elongated pleomorphic nuclei (Figure 3C). Immunostaining revealed that the tumor cells were strongly positive for CK20 (Figure 3D) and CDX2 (Figure 3E), whereas the endometrial glands and stroma did not react with CK20 or CDX2. CK7 expression was completely absent in the tumor cells; however, it was uniformly positive in the endometrial glands and stroma. In addition, infiltrating tumor cell clusters associated with small amount of stromal desmoplasia and artifactual clefts (Figure 3F) were seen. Compared with atrophic or inactive endometrial glandular epithelium, the tumor cells possessed large, hyperchromatic nuclei (Figure 3G).

Metastatic signet ring cell carcinomas of gastric (case 6) and appendiceal (case 7) origin. Several large endometrial tissue fragments contained densely aggregated tumor cells. The tumor cell sheets replaced the endometrial stroma and entrapped benign endometrial glands (Figure 3H). The tumor cells were large and round, and possessed abundant intracytoplasmic mucin, that displaced the nucleus to one side of the cell (Figure 3I). The nuclei were atypical and crescent-shaped, showing prominent nucleoli and frequent mitotic figures. Typical morphological features of signet ring cells, i.e., large collections of intracytoplasmic mucin compressing the nuclei towards the periphery of the cell, were present (Figure 3J). The metastatic tumor tissue almost exclusively comprised signet ring cells. Although the tumor cells were arranged in large irregular clusters, those in some foci were scattered singly in the endometrial stroma. The tumor cells did not express ER, PR, and PAX8.

## 4. Discussion

Secondary tumors of the uterus are classified into two major groups, namely those of genital and extragenital organs. The uterine body and cervix are frequently involved by direct extension from pelvic or extragenital tumors. Malignancies of neighboring organs, including the ovaries, salpinges, urinary bladder, and rectum, may metastasize to the uterus via lymphatics or blood vessels; however, most cases represent direct local extension. Uterine metastases from extragenital malignancies are rare, and account for less than 10% of all cases of metastases to the female genital tract [10,15]. Carcinomas of the breast and gastrointestinal tract and are the most frequent extragenital malignancies to metastasize to the uterus. Approximately 200 cases of uterine metastases have been documented in the literature; many of them are from autopsy series, and recognize a mammary origin [8,9]. Other primary tumors include carcinomas of the lung, kidney, urinary bladder, and pancreas, and cutaneous malignant melanomas, soft tissue sarcomas, and medullary thyroid carcinomas [9,10].

Metastatic tumors to the uterine body usually involve the myometrium; those exclusively involving the endometrium are rare. Endometrial involvement has been reported from breast carcinoma [13], colorectal carcinoma [3,7], gastric carcinoma [6], and malignant melanoma [2,4,31]. In cases of endometrial involvement, abnormal uterine bleeding appears to be the first presenting sign of metastasis [13,31]. This presents simultaneously, prior to, or following the diagnosis of the primary tumor. Endometrial metastasis is associated with disseminated spread of cancer, and eventually shortens life expectancy [5,13,32]. In cases of abnormal uterine bleeding in patients with a history of extrauterine malignancies, histological examination should be performed to ensure appropriate diagnosis and treatment. More importantly, endometrial curettage should be performed with extreme caution because the procedure may lead to uncontrollable hemorrhage in patients with myometrial involvement by the metastatic tumor.

In this study, we described the clinicopathological characteristics of extragenital malignancies that had metastasized to the endometrium, with features mimicking primary endometrial carcinomas. Notably, this series is probably the first to describe the occurrence of endometrial metastasis from nasopharyngeal carcinoma. To the best of our knowledge, distant metastasis of nasopharyngeal carcinoma to the endometrium has never been documented in the literature. Although the majority of nasopharyngeal carcinomas present in localized stages, and local therapy may be curative, the potential for developing distant metastases is high. It usually metastasizes to the liver, lung, and bone, via both, hematogenous and lymphatic routes [33,34,35]. Reports suggest that approximately half of the metastatic lesions are detected within a year after completion of radiation therapy. The median survival of patients who developed bone metastases from nasopharyngeal carcinoma was found to extend for less than a year [34,35]. The accurate discrimination between primary endometrial lymphoepithelioma-like carcinoma (LELC) and metastatic nasopharyngeal carcinoma is a major issue. The former is extremely rare, and only four cases have been reported [36,37,38]. It is difficult to distinguish between the two lesions morphologically, as both display multinodular growth, a syncytial growth pattern of tumor cells with an admixture of inflammatory cellular elements, high-grade nuclear atypia, and increased mitotic activity. Furthermore, although the identification of EBV has been established as a useful diagnostic marker of nasopharyngeal carcinoma, one of the four previously reported cases of endometrial LELC had tested positive for EBER-ISH, indicating that detection of EBV does not definitely exclude the possibility of primary endometrial LELC. Therefore, to distinguish metastases from primary endometrial tumors, pathological and immunohistochemical findings should be considered in conjunction with the patient’s clinical history. Uterine metastases from nasopharyngeal carcinoma are very rare, and typically occur in association with disseminated disease. In the clinical context, the previous diagnosis of nasopharyngeal carcinoma alone indicated the metastatic nature of the endometrial lesion in our patient with multiple metastatic lesions in the extra-uterine organs.

Breast carcinoma is the most common extragenital malignancy to metastasize to the endometrium [13]. Endometrial metastases from breast cancer tend to occur together synchronously with multi-organ metastases [5,13]. Despite treatment with adjuvant chemoradiation therapy after surgical resection, breast cancer is known to have high metastatic potential [5,13,32,39,40]. Among breast carcinomas, the invasive lobular type is most frequently associated with endometrial metastasis. This may be attributed to the fact that invasive lobular carcinoma has a greater propensity to metastasize to the peritoneal and retroperitoneal organs than other types of breast carcinoma [13,40]. In this study, one of the two cases of breast carcinoma had a histological diagnosis of invasive lobular carcinoma, and demonstrated multiple metastases to other organs. Since both, metastatic invasive lobular carcinoma of the breast and primary endometrial carcinoma demonstrate positivity for ER and PR, immunostaining for hormone receptors is not helpful in distinguishing tumors of metastatic mammary origin from those of primary endometrial origin. Pathologists may alternatively employ PAX8 (for endometrial origin), gross cystic disease fluid protein 15 (GCDFP15), mammaglobin, and GATA3 (for mammary origin) for the differential diagnosis. One of the cases in this series had metastatic invasive ductal carcinoma; endometrial metastasis is relatively uncommon with this histological type. Moreover, the tumor cells tested negative for ER, PR, and HER2; this was suggestive of TNBC. Endometrial metastases from TNBC are extremely rare, with only two reported cases [13,41]. High-grade endometrial carcinomas such as grade 3 endometrioid and serous carcinomas may be included in the differential diagnosis of metastatic TNBC. Since these endometrial tumors are negative or weakly positive for hormone receptors, immunostaining for PAX8 is more helpful for obtaining an accurate diagnosis. In a subset of breast carcinomas that exhibit high-grade nuclear atypia, and TNBCs, the expressions of breast-specific markers such as GCDFP15, mammaglobin, and GATA3 may be negative or weakly positive.

Mazur et al. [10] analyzed 56 cases of colorectal carcinoma with metastases to the female genital tract; only 2 (3.6%) cases in that cohort demonstrated endometrial involvement. Furthermore, only two case reports of endometrium-limited metastasis from colonic adenocarcinoma are available in literature [3,7]. In these case reports, there was no evidence of metastasis to other sites; concurrent primary endometrial carcinoma of the endometrioid type was identified in one case. In our case, the clinical history of disseminated metastases provided the clue for considering the possibility of metastatic carcinoma to the endometrium. Histological distinction between metastatic colonic adenocarcinoma and primary endometrial endometrioid carcinoma is particularly challenging owing to similarities in their morphological features. The former is frequently associated with poor cytoarchitectural correlation, high-grade nuclear atypia, and intraluminal dirty necrosis. Our cases demonstrated poorly differentiated carcinoma with frequent intraluminal necrotic debris. Since grade 3 endometrioid carcinoma, serous carcinoma, and poorly differentiated carcinomatous components of carcinosarcoma may also display severe nuclear pleomorphism and coagulative tumor cell necrosis, combined immunostaining for CK7, CK20, and CDX2 is particularly useful for the differential diagnosis [42]. The lack of CK7 expression and uniform immunoreactivity for CK20 and CDX2 supports a diagnosis of metastatic carcinoma of colorectal origin.

Among carcinomas of the gastrointestinal tract, appendiceal primaries are rare [43,44]. Moreover, appendiceal signet ring cell carcinoma is an extremely rare malignancy, that accounts for 4% of all appendiceal tumors [45]. The most common metastatic sites of appendiceal carcinoma are the ovaries and peritoneum [12]. Mazur et al. [10] reported a single case of an appendiceal tumor that had simultaneously metastasized to the ovary and endometrium; however, its histological features had not been mentioned. Pan et al. [46] reported a case of metastasis of the signet ring cell component of a goblet cell carcinoid of the appendix to the endometrium. To the best of our knowledge, our case series is the first to report a case of endometrial metastasis from a pure signet ring cell carcinoma of the appendix.

Similar to other gastrointestinal tumors, gynecological metastases from gastric carcinoma have a predilection for the ovaries. Mazur et al. [10] reported eight cases of metastatic carcinoma from the stomach to the female genital tract. In six of the eight cases, the site of metastasis was the ovaries, and in the remaining two, the sites were the endometrium and uterine cervix. Three individual case reports have been published regarding metastases of gastric carcinoma to the uterine corpus [6,47,48]. Histologically, the tumors were carcinomas of the diffuse and anaplastic types. Two of the three cases were diagnosed from endometrial biopsy or curettage specimens. The other case was diagnosed after hysterectomy; it demonstrated diffuse lymphovascular invasion within the myometrium. In our case, metastases to the peritoneum, omentum, and perigastric lymph nodes were present prior to the detection of the endometrial lesion. Nevertheless, our finding was uncommon in that the endometrium was the only metastatic site in the uterus, and the myometrium and bilateral adnexae were free of carcinoma.

The presence of signet ring cells is an uncommon finding in primary endometrial carcinomas. In cases where the signet ring cells are accompanied by an endometrioid carcinoma component, the tumor may be diagnosed as an endometrioid carcinoma with signet ring cells [49]. Primary endometrial signet ring cell carcinoma, in which the entire or majority of tumor tissue consists of signet ring cells, is even rarer, and only two cases have been reported [49,50]. In cases where signet ring cell components are identified in endometrial tumors, metastases from extragenital organs should be initially considered. The absence of precursor lesions, diffuse myometrial infiltration, and extensive lymphovascular invasion are suggestive of metastatic tumors [49,50]. Immunostaining may be helpful in differentiating between primary endometrial and metastatic tumors, particularly in cases where tumor cells demonstrate signet ring cell-like morphology. Primary endometrial lesions test positive for PAX8, ER, and vimentin, whereas those of gastric or appendiceal origin test negative.

In summary, we investigated the clinicopathological characteristics of seven cases of endometrial metastasis from extragenital malignancies. Secondary tumors of the uterine body almost always involve the myometrium; therefore, the prediction of endometrium-limited metastasis from curettage specimens is particularly challenging. The possibility of endometrium-limited metastasis should be considered in patients with a previous history of extrauterine malignancies, and in those with widespread metastases. Pathological examination and knowledge of the clinical history may help differentiate between primary and metastatic carcinomas. Metastases to the endometrium from extrauterine malignancies usually indicate disseminated disease and a poor prognosis. The accurate diagnosis of metastases is therefore essential to ensure proper management. Cumulative evidence from further reports will be needed to validate our findings.

## Figures and Tables

**Table 1:** Clinical characteristics of the patients.

Case No.	Age (Years)	Previous Gynecological History	Presenting Symptom	Site of Primary Tumor	Histology of Primary Tumor	Location of Uterine Metastasis	Location of Extrauterine Metastasis	Treatment	Current Status
1	44	None	Abnormal uterine bleeding	Nasopharynx	Non-keratinizing undifferentiated carcinoma	Endometrium only	Ovary, lymph node, lung, liver, bone, adrenal gland, retroperitoneum	Chemotherapy	Progressive disease
2	60	None	Abnormal uterine bleeding	Breast	Invasive lobular carcinoma	Endometrium only	Ovary, lymph node, liver, bone, retroperitoneum	Chemotherapy	Progressive disease
3	47	None	Abnormal uterine bleeding	Breast	Invasive ductal carcinoma	Endometrium only	Lymph node, lung, scalp	Chemotherapy	Progressive disease
4	71	None	Abnormal uterine bleeding	Colon	Adenocarcinoma	Endometrium only	Lung, liver, bone, omentum, abdominopelvic peritoneum	Chemotherapy	Dead of infection
5	67	None	Abnormal uterine bleeding	Colon	Adenocarcinoma	Endometrium only	Ovary, lung, liver, abdominal wall	Chemotherapy	Dead of carcinoma
6	63	None	Abnormal uterine bleeding	Appendix	Signet ring cell carcinoma	Endometrium only	Ovary, abdominopelvic peritoneum	None (patient refusal)	Follow-up loss
7	36	None	Abnormal uterine bleeding	Stomach	Signet ring cell carcinoma	Endometrium only	Lymph node, omentum, abdominopelvic peritoneum	Chemotherapy	Progressive disease

## Abbreviations

FFPE	Formalin-fixed, paraffin-embedded
EBV	Epstein-Barr virus
EBER-ISH	Epstein-Barr virus-encoded RNA in situ hybridization
CK	Cytokeratin
CDX2	Caudal type homeobox 2
PAX8	Paired box 8
ER	Estrogen receptor
PR	Progesterone receptor
GATA3	GATA-binding protein 3
CT	Computed tomography
MRI	Magnetic resonance imaging
PET-CT	Positron emission tomography-computed tomography
HER2	Human epidermal growth factor receptor 2
TNBC	Triple-negative breast carcinoma
LELC	Lymphoepithelioma-like carcinoma
GCDFP	Gross cystic disease fluid protein 15
